# Unravelling the epidemiology of late-onset and asymptomatic carriers of FAP ATTR V30M in a Portuguese population

**DOI:** 10.1186/1750-1172-10-S1-O3

**Published:** 2015-11-02

**Authors:** Carolina Lemos, Teresa Coelho, Miguel Alves-Ferreira, Diana Santos, Jorge Sequeiros, Alda Sousa

**Affiliations:** 1 IBMC and ICBAS, UnIGENe, 4150-180, Porto, Portugal; 2 Centro Hospitalar do Porto, Unidade Corino de Andrade, 4050 - 345, Porto, Portugal

## Background

Familial Amyloid Polyneuropathy (FAP ATTRV30M) is an AD systemic amyloidosis, due to a point mutation in the transthyretin (TTR) gene. Although in Portugal the disease has been characterized by its early onset (lower than 40yrs), a wider age-at-onset (AO) variability has been uncovered. The mean AO is 35.3, but more and more late-onset (higher than 50yrs) cases are being ascertained, often matched with older asymptomatic parents. Our aim now was to look into late-onset cases and aged-asymptomatic carriers in order to unravel familial aggregation of late-onset and to characterize these families regarding their epidemiology.

## Methods

From the largest registry worldwide with 2754 patients (678 families), we analyzed a group of 326 late-onset cases (133 families) regarding gender and also their transmitting parent. Additionally we analyzed 222 asymptomatic carriers on regular follow-up, aged above 40 at last observation and their first-degree relatives, belonging to 122 families. We performed a descriptive analysis and used the Student's t-test for comparisons between groups.

## Results

Age-at-onset was 60.03 for men and 59.25 for women (NS), as opposed to the general sample where women had a later onset (37.6) than men (33.4). Familial aggregation of late-onset cases is apparent, with some families having up to 11 late-onset cases. Out of 678 probands, ~40% had no affected parent at time of diagnosis, this figure being 86% (115/133) among late-onset probands. These parents had died with no signs of the disease mostly at old-age. No one had an affected parent with early-onset of the disease.

For asymptomatic carriers, age-at-last-observation varies between 40 and 49 for 103 subjects and was above 50 for 119 of them. Mean age-at-last-observation was 54.06 (SD: 12.2; range: 40-89) and no gender differences were found. We were able to identify 92 transmitting-parents (59 fathers, 33 mothers) with know AO. Their mean AO was 56.91 (SD: 12.8; range: 25-80) and no differences in AO were found between parent's gender. Also, we found a mean AO close to 40 years for siblings of these asymptomatic carriers (mean: 39.91; SD: 8.89; range: 24-65).

## Conclusions

While most of FAP probands had one affected parent (as expected in an AD disease), a significant number has a late-onset and no affected parent at time of diagnosis. We confirmed familial aggregation of late-onset cases. We also found that for late-onset cases no gender differences are observed. This shows that some families are protected from the severe manifestations of FAP. Due to these different clinical aspects of FAP in late-onset patients it is crucial to explore mechanisms that can be related with aging and protective factors that can lead to new therapeutic strategies.

